# Advancing vision: gene therapy innovations for X-linked retinitis pigmentosa (XLRP)

**DOI:** 10.17179/excli2025-8593

**Published:** 2025-08-08

**Authors:** Md Sadique Hussain, Amita Joshi Rana, Janaki Ramaiah Mekala, Purushothaman Balakrishnan, Sivakumar Arumugam, Gaurav Gupta, Prasanna Srinivasan Ramalingam

**Affiliations:** 1 Uttaranchal Institute of Pharmaceutical Sciences, Uttaranchal University, Prem Nagar, Dehradun 248007, Uttarakhand, India; 2 School of Pharmaceutical Sciences, Lovely Professional University, Phagwara 144411, Punjab, India; 3 College of Pharmacy, Graphic Era Hill University, Bhimtal, Uttarakhand 263136, India; 4 School of Biosciences and Technology, Vellore Institute of Technology, Vellore, India; 5 Department of Biomaterials, Saveetha Dental College and Hospitals, SIMATS, Saveetha University, Chennai-600077, India; 6 Centre for Research Impact & Outcome-Chitkara College of Pharmacy, Chitkara University, Rajpura, Punjab 140401, India; 7 Centre of Medical and Bio-allied Health Sciences Research, Ajman University, Ajman, UAE

## ⁯⁯⁯

X-linked retinitis pigmentosa (XLRP) represents one of the most severe forms of inherited retinal degeneration, predominantly caused by mutations in the RP GTPase regulator (RPGR) gene (Parmeggiani et al., 2025[8]). These mutations lead to progressive photoreceptor cell loss, culminating in visual impairment and eventual blindness (Mansouri, 2023[6]). Over the past decade, advances in gene therapy have paved the way for promising interventions targeting the molecular basis of XLRP.

Recent preclinical studies utilizing CRISPR-Cas9 technology and adeno-associated viral (AAV) vectors have demonstrated significant progress. In mouse models with RPGR-related XLRP, targeted editing of the RPGR gene using single-guide RNA (sgRNA) in combination with CRISPR-Cas9 has shown potential in preserving photoreceptor integrity. Notably, subretinal injection of CRISPR-Cas9 AAV vectors has successfully restored the open reading frame (ORF) of RPGRORF15, achieving widespread retinal distribution and functional recovery in rd9 mice (Hu et al., 2020[3]; Martinez-Fernandez De La Camara et al., 2018[7]). These findings underscore the therapeutic viability of precise gene-editing tools for retinal disorders.

Building upon these preclinical successes, several clinical trials have been launched to assess the safety and efficacy of RPGR-targeted gene therapy in humans (Gumerson et al., 2022[2]). One notable effort is the phase I/II clinical trial (NCT03116113) employing BIIB112, an AAV vector encoding codon-optimized human RPGR (AAV8-coRPGR). In this dose-escalation study, a single subretinal injection was administered to individuals with XLRP caused by RPGR mutations. While higher doses were associated with steroid-responsive subretinal inflammation, encouragingly, six patients experienced sustained improvements in their visual field, observed as early as one-month post-injection and persisting through follow-up assessments (Cehajic-Kapetanovic et al., 2020[1]).

Another pivotal trial involves the rAAV2-RPGR vector, aimed at addressing faulty RPGR in both pediatric and adult patients with XLRP. This phase I/II dose-escalation trial (NCT03252847) has been completed, and the results are eagerly awaited. Meanwhile, the phase I/II trial evaluating rAAV2tYF-GRK1-RPGR (NCT03316560) continues to explore safety and efficacy parameters. Complementing these efforts is a phase III study dedicated to assessing AAV5-hRKp.RPGR in Japanese patients with RPGR mutations (NCT05926583). Furthermore, a phase 3 study (NCT04671433) evaluating the safety and efficacy of botaretigene sparoparvovec (AAV5-RPGR), an investigational gene therapy for XLRP. The therapy aims to deliver a functional copy of the RPGR gene to retinal cells using an AAV vector. Together, these studies represent a comprehensive global effort to validate gene therapy as a transformative treatment for XLRP.

The broader implications of gene therapy extend beyond reversing retinal damage. By addressing the underlying genetic cause, these therapies have the potential to restore functional vision and improve quality of life significantly (Hussain et al., 2025[4]). Importantly, the ability to deliver a therapeutic gene using AAV vectors has revolutionized the treatment landscape for inherited retinal diseases, offering targeted delivery with minimal off-target effects.

Despite these advancements, challenges remain. One significant hurdle is the variability in patient response, influenced by factors such as mutation type, disease stage, and individual immune responses to AAV vectors. Moreover, long-term safety data are critical, particularly regarding potential immunogenicity and off-target effects of gene-editing technologies like CRISPR-Cas9 (Hussain et al., 2025[5]). Addressing these challenges will require continued refinement of vector design, dose optimization, and patient stratification strategies.

As the field advances, innovations such as dual-vector systems, promoter engineering, and non-viral delivery platforms are likely to further enhance the precision and effectiveness of gene therapy for XLRP. Moreover, integrating gene therapy with other emerging modalities, such as optogenetics and cell-based therapies, could unlock new therapeutic avenues. Such combinatorial approaches hold the promise of not only halting disease progression but also restoring complex visual functions in patients with advanced retinal degeneration.

Looking ahead, the future of gene therapy for XLRP appears significantly promising. However, continued efforts such as development of potential delivery methods, establishing long-term safety, and tailoring treatments towards individual patients will pave the way for precision treatment in XLRP. Additionally, integrating these novel gene therapies with regenerative therapies may further offer possibility for restoring vision and improving XLRP patient lives.

Gene therapy for XLRP has transitioned from preclinical promise to clinical reality, offering hope to individuals afflicted by this devastating condition. The strides made in understanding the molecular mechanisms of RPGR mutations have catalyzed the development of targeted therapies capable of altering disease trajectories. As ongoing clinical trials yield data, the integration of these findings into broader treatment frameworks will define the future of personalized medicine in ophthalmology. With continued innovation, gene therapy is poised to transform the management of XLRP, paving the way for broader applications in other retinal and genetic disorders.

## Declaration

### Author contributions 

Md Sadique Hussain was involved in conceptualization, and all the other authors involved in data curation and writing - original draft, and writing - review and editing. All authors have approved the final version of the manuscript.

### Ethics approval and consent to participate 

This article was not subject to any ethics approval from the institutions. All authors provided their consent to participate. 

### Consent for publication 

All authors gave their consent for publication of this article. 

### Declaration of competing interest 

The authors declare that they have no known competing financial interests or personal relationships that could have appeared to influence the work reported in this paper. 

### Declaration of Generative AI and AI-assisted technologies in the writing process

During the preparation of this work, the author(s) used ChatGPT to correct the grammatical and typographical errors in the manuscript. All authors have read and approved the final version of the manuscript.
